# Management of Menstrual Hygiene in Promoting Education Among Adolescent Girls in Enqucwini Education Zone in Mzimba District

**DOI:** 10.1002/puh2.70362

**Published:** 2026-09-15

**Authors:** Sellah Vitumbiko Nkowani, Benard C. G. Kamanga, Zondiwe Chingati, Thokozani Mzumara

**Affiliations:** ^1^ Department of Nursing and midwifery Mzimba North District Health Office Mzuzu Malawi; ^2^ Department of Public Health University of Livingstonia Mzuzu Malawi; ^3^ Optometry unit Department of curative and rehabilitative services Mzuzu Urban Community Hospital Mzimba North District Health Office Mzuzu Malawi

**Keywords:** adolescent, hygiene, Malawi, menarche, menstrual cycle, menstruation, rural, sanitation, schools

## Abstract

**Background:**

Poor menstrual hygiene management (MHM) greatly impacts girls’ education negatively—however, studies exploring menstrual health management in sub‐Saharan rural region are rare.

**Aim:**

The study explored MHM practices among adolescent girls in rural Mzimba, ⁴ Malawi.

**Method:**

It was a mixed‐methods study with a sample size of 81. Data were collected through questionnaires, direct observations, FGDs, key informants and participant observation that were used to collect the experiences. Qualitative data were analysed using a thematic approach, and quantitative data using SPSS.

**Findings:**

The study results showed that common sanitary facilities were pit latrines (100%), no bathroom during the study period (0%) to be used to bathe and change. Further, the study found that lack of education on menstrual management (96%) and 63% of the respondents cited lack of adequate facilities. The study found lack of water buckets at the toilets (28.1%), and about 23.1% said they did not wash hands because of a lack of handwashing soap. However, the majority of the participants explained that hand washing was not done because of both reasons: lack of water buckets and soap for hand washing (48.8%). Cultural practices promoted absenteeism among young adolescent girls who are prevented from attending school during menstruation to avoid shaming the family.

**Conclusion:**

The study recommends compliance with the sanitation policy, that is, school management to invest in the development of facilities and promotion of menstrual hygiene practices through education to curb harmful cultural practices related to menstruation.

## Graphical abstract

Poor Menstrual Hygiene Management (MHM) greatly impacts girls’ education negatively. The study results showed that common sanitary facilities were pit latrines (100%), and no bathroom was used to bathe and change. The majority of the participants explained that hand washing was not done because of two reasons: lack of water buckets and soap for hand washing (48.8%).

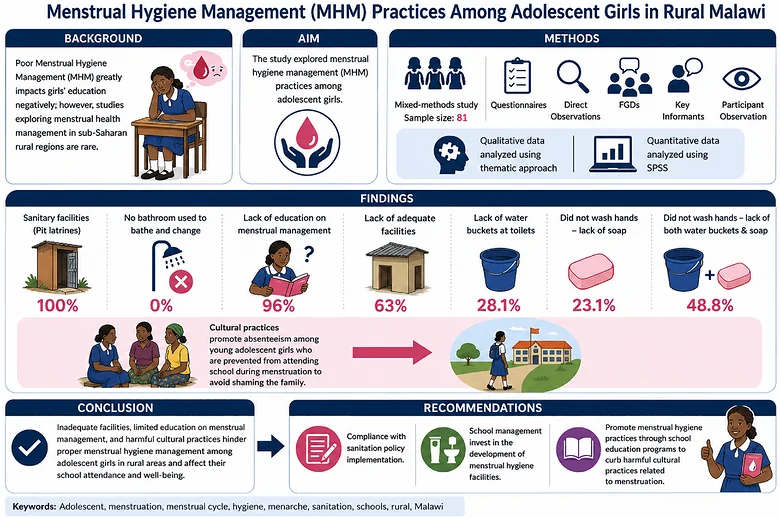

## 1 Introduction

Menstrual hygiene management (MHM) has become a globally recognised public health issue. Menstruation is a biological activity every girl and woman experiences. It is the shedding of the endometrium as a result of actions by female‐dominating hormones—the oestrogen hormones.  MHM encompasses a wider area, including availability of places to wash, choice of material, disposal and emerging technologies, including availability and knowledge in particular social contexts with regard to taboos and stigma associated with menstruation [1]. Despite various interventions aimed at improving MHM products and education, cultural taboos and limited knowledge further act as hindrances, restricting girls’ ability to manage menstruation with dignity, thereby impacting their education and empowerment (Enzler et al. 2018). In Malawi, the availability of appropriate places for washing during menstruation is inadequate, particularly in schools and rural communities [2]. This insufficiency hampers effective MHM and contributes to increased absenteeism and potential health risks among adolescent girls and women. Evidence indicates that inadequate sanitation facilities and poor access to water for washing during menstruation affect the health, well‐being and school attendance of girls and women [3].

Although improvements in gender equality in education have occurred  with the number of girls for every 100 boys in primary education rising from 92 to 97 and from 91 to 97 in secondary education, there were still many challenges that pushed girls out of education. Globally, girls’ education is as important as that of boys because they have the same right to education as boys. This is why there is total commitment by the world to continue making progress through the United Nations Sustainable Development Goals (SDGs). Goal 4 aims to ‘ensure that all girls and boys complete free, equitable and quality primary and secondary education’. Goal 5 is focused on ‘achieving gender equality and empowering all women and girls’.

Many studies have shown that girls lacked the right information and support about menstruation, and this largely contributes to poor MHM [4]. This has contributed to school absenteeism and school dropout of girls [5]. The poor sanitary facilities and the lack of knowledge on menstruation influenced girls’ education, as it resulted in school absenteeism during menstruation, and later, others dropped out of school because such girls found it difficult to manage their menses in a dignified manner due to compromised sanitary facilities. The long absenteeism from school renders it difficult for such girls to cope with the boys’ performance. These experiences hinder the attainment of education by girls, and such girls failed to realise their education [5].

Poor sanitary facilities contributes to poor MHM, which had several contributing factors to girls’ education. The experiences girls encounter during menstruation and the lack of the right information (individual) made the girls absent from school and opt to nurse menses at home. Lack of proper sanitary facilities and sanitary resources facilitates school absenteeism during menstruation. The lack of support, such as policy implementation by the education managers (organisation), by providing necessary sanitary resources, could have improved the MHM, which could help to contain girls at school during menstruation and prevent absenteeism and school dropout.

Many studies around the world have shown that girls lack the right information to support menstruation, and this largely contributes to poor MHM. This has been shown to contribute to school absenteeism and school dropout of girls [5]. Furthermore, a lack of the right information and a lack of sanitary facilities result in girls remaining ignorant. Moreover, the lack of necessary menstrual resources due to poverty forces some girls to practise unhygienic ways of managing menstruation, which results in adverse health outcomes, as some girls got sick from Urinary Tract Infections (UTIs) and miss classes during menstruation. Additionally, others indulge in sexual activities in exchange for sanitary pads, which eventually contract Sexual Transmitted Diseases (STD). This was evidenced by the study, which was conducted in India, Singur, West Bengal community [6].

Previous studies conducted in Malawi focused mainly on the central [7] (Alva Vilda et al. 2022) and southern region [8] (Roxburg et al. 2020), often neglecting the northern region. Only one study was conducted in the northern region, focusing on an urban setting [9]. Nevertheless, to the best of our knowledge, no study has been conducted in northern rural settings on menstrual hygiene practices among adolescent girls. Notably, the majority of the Malawian population resides in the rural areas where resources, including information, are constrained compared to the urban settings [10]. Therefore, this study aimed at exploring the MHM practices among adolescent school girls in Enguqwini Education Zone in rural Mzimba District. Specifically, the study assessed the availability and characteristics of sanitary facilities that support management of menstrual hygiene among learners at the school. The findings of the study will aid planners and policymakers in implementing strategies and tactics to promote MHM among girls and improve dignity and the empowerment of the girl child in the setting.

## 2 Methods

### 2.1 Study Design

The study employed a mixed approach that employed an explanatory sequential design where quantitative was collected first then qualitative data. This approach allowed the researcher to first gather numerical data and then use qualitative data to enhance and validate the findings [11]. By incorporating both approaches, the study aimed to overcome the limitations of each and provide a comprehensive understanding of the phenomena under investigation. Although quantitative research adds a numerical dimension and improves the accuracy of research instruments, qualitative research allows for a contextualised analysis of lived experiences.

### 2.2 Study Setting

**FIGURE 1:**
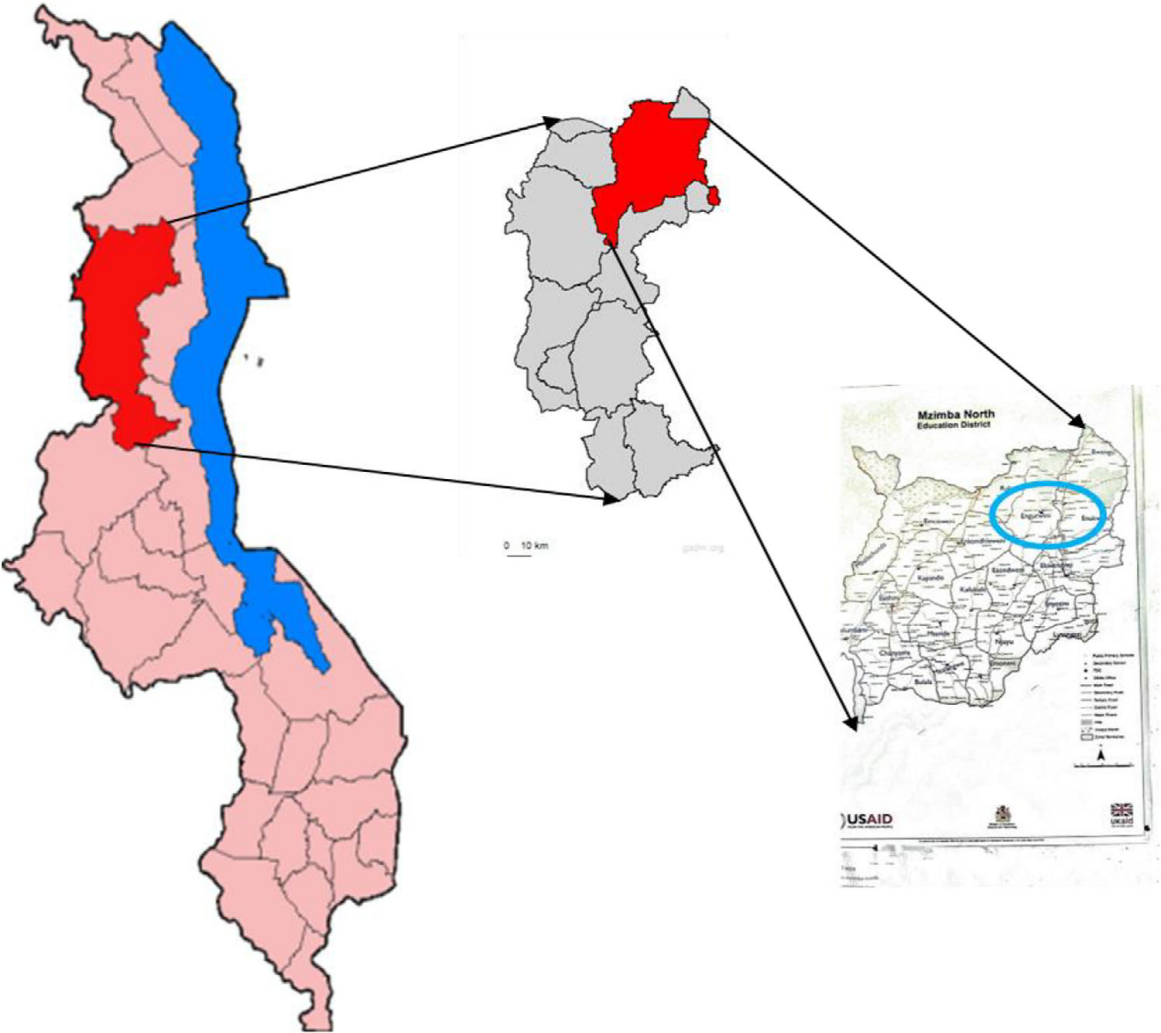
Map of Malawi, isolating the study area Enqucwini.

The study was conducted within the Enqucwini Education Zone in Mzimba North District, mainly at Chilukwa (Figure 1). About 11°54′0″ S, 33°36′0″ E Latitude position: Equator = 1323 km (822 mi) = Mzimba = 8684 km (5396 mi) = south [12].

### 2.3 Study Population

The study audience consisted of the head master, teachers, and adolescent girls from Chilukwa Primary School and female community members of the Mother Care Groups (MCG) who were included in qualitative part.

The quantitative part consisted of adolescent girls only.

### 2.4 Inclusion and Exclusion Criteria

The study included female adolescent girls aged between 10 and 15. Furthermore, the study included girls who had attained menarche.

### 2.5 Sampling Technique

The study employed random sampling technique to select adolescents for the quantitative analysis, whereas the qualitative part participants were selected purposively.

### 2.6 Data Collection

The researcher also interviewed the women from MCGs, who represented the community members as key informants. This group of women comprised women from different villages and different ages, but mostly elderly. This type of grouping was responsible for health and education‐related issues in the community. It was the existing group for advising the girls when they attained menarche and subsequent menses on menstrual management in the community and had information about the culture, which was also shared with adolescents.

The questionnaire was pilot tested at another school in the district. Content validity was achieved through review by experts familiar with the subject area. The content of the questionnaire was aligned with the study aim of the article. Accordingly, the study qualitative questionnaire focused on the following aspects: practices and behaviours related to menstruation in the school setting and cultural practices related to menstruation that affect education among adolescent girls. The quantitative part focused on the availability of sanitation facilities that would support menstrual hygiene practices, such as availability of hand washing facilities, characteristics of toilets, availability of changing rooms and availability of policy documents.

### 2.7 Sample Size Determination

The sample size for qualitative data was based on the availability of information until information saturation was reached. The study involved 2 focus group discussions from Chilukwa Primary School with 8–10 girls in each FGD.

For the quantitative part, the equation was developed by Cochran in 1963 and adopted by Israel (Eshetu et al. 2026). This formula is used if the population is not known to determine the sample size as follows:

$$n_{0}=\frac{Z^{2}pq}{e^{2}}$$

where $n_{o}$ is the sample size;$\;Z^{2}$ is the confidence level of 95% (1.96); *p* is the estimated proportion of an attribute that is present in the population; *q* is 1 − *p*, which is 1 − 0.5 = 0.5; and $e^{2}$ is the error margin of the width of the confidence level, which is 0.109.

Using a calculator, the sample size was computed as follows:

$$n_{0}=\frac{Z^{2}pq}{e^{2}}$$

$n_{0}$ = 1.96^2^ × 0.5 × 0.5/0.109^2^

 = 80.8349

Therefore, the sample size is 81.

### 2.8 Data Analysis

Qualitative data, which were audio recorded, were translated and transcribed before being engaged in thematic analysis. The quantitative data were entered into SPSS version 27. Descriptive statistics, including frequency and percentage, were employed. We utilised tables to graphically illustrate the data.

### 2.9 Ethical Considerations

The UNILIA‐REC under the University Research Department gave clearance to conduct the research in these schools (reference number: UNILIA‐REC/PGS/01/23) on fourth September 2023 and adhered to the Declaration of Helsinki. The researcher got consent from the parents/caregivers of each participant since the participants were minors. Next, all research documents were kept in a locked cabinet and had no names of participants or any identifiers that might lead to the participants. Participants joined the study of their own free will and were not coerced into doing so. In addition, the participants were allowed to withdraw at any time during the study if they so wished. Participants were assured that dropping from the study would not lead to any punitive measures or negative impacts. Confidentiality of information was maintained by excluding any personal identifiers, and the recorded data were stored in a safe place where no one except study staff accessed the results.

## 3 Results

### 3.1 Demographic Information of the Respondents

The results show that the majority of respondents were adolescent girls aged between 10 and 15 years, followed by those in the age range of 16–25 years, implying the likelihood of the existence of challenges in MHM. The results indicated that most of the respondents were in the category of senior primary (from Standard 5 to Standard 8).

The 28% of respondents were teachers, mostly females. The study interviewed fewer men on issues of menstruation to understand their position on the same and also interviewed the members of the MCG as custodians of culture.

### 3.2 Availability of Sanitary Facilities

Most of the sanitary facilities available at Chilukwa school were pit latrines.

There were 3 pit latrines functioning against 315 girls at the school. The toilets did not provide privacy and dignity to girls as they did not have doors, hence compromising privacy and dignity. The toilets were not very far from the boys’ toilets, and the boys were passing by girls’ toilets to their toilets.

**FIGURE 2:**
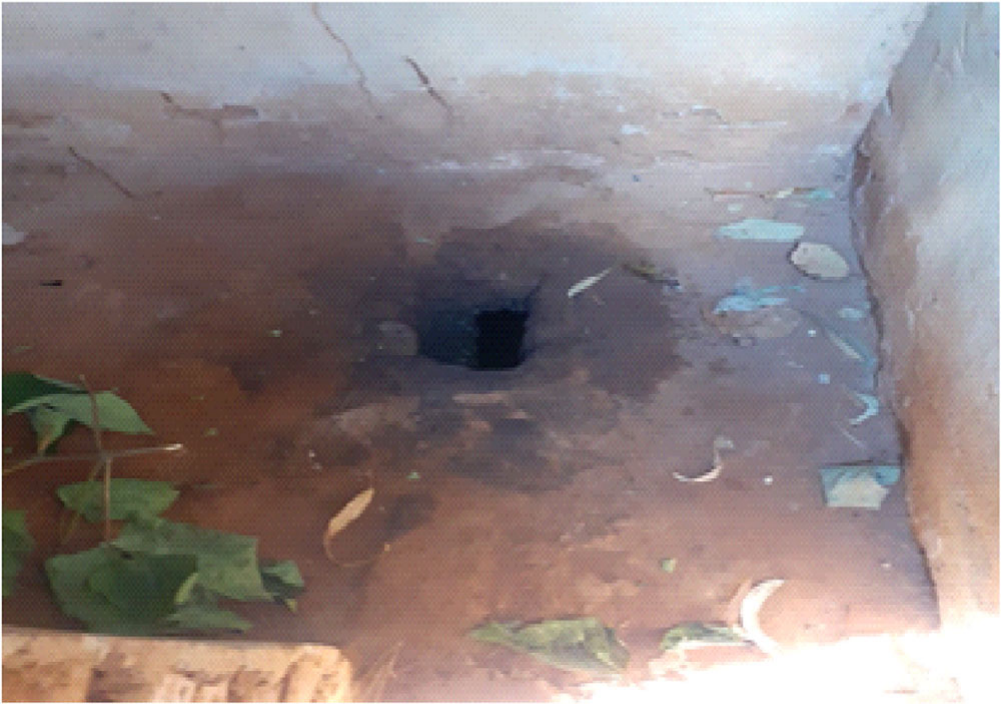
A toilet without a drophole cover.

**FIGURE 3:**
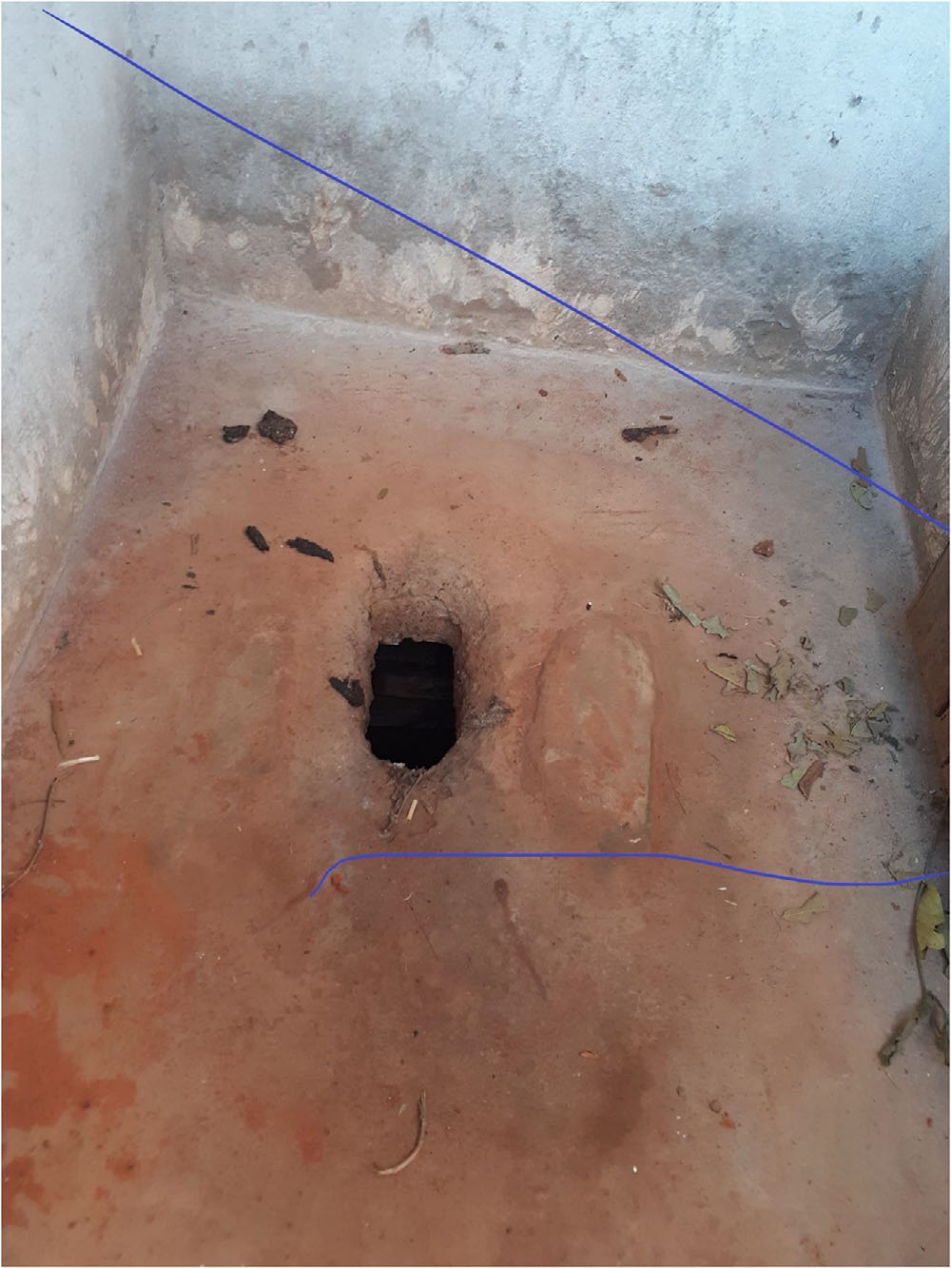
A dirty toilet with no drophole cover at Chilukwa Primary School in 2023.

From the observation done during the visit to Chilukwa School, the toilets had no drop hole covers (Figures 2 and 3).

The main reason given for not covering the toilet holes was a lack of materials to cover/make drop hole covers (56%). This was followed by an ignorance (30%) on the importance of covering the toilets.

Chilukwa Primary School had no bathroom and an incinerator to throw the soiled pads and bath when soiled.

Total pit latrines were 3, which were functional against 315 girls and 5 for boys from Chilukwa School. The toilets were very dirty with no drop hole covers, and there were faeces all over the toilet floor, and others practised open defecation outside the toilets.

The pupils were not washing their hands after using the toilets. This was due to several reasons, and the main reason from the results was a lack of both water buckets and soap for hand washing (48.8%), no water buckets was 28.1%, and those participants who responded no soap for hand washing were at 23.4%.

Rubbish pits for waste management were available, representing 95.5%. Some girls also used the rubbish pits for throwing the soiled pads/cloths. At Chilukwa, the full rubbish pits were evacuated, and the contents were used as manure in a demonstration land. Sometimes the school planted bananas in the full pits for consumption. The majority of the pupils used the pits to throw waste (84%), and a small portion threw soiled pads (15%).

The majority of the respondents (78%) had no idea of the availability of guidelines, which included MHM, and 22% had an idea of the availability of the guidelines. At Chilukwa, these guidelines were not available at all, and the headmaster also indicated no idea of their availability.

There were several reasons for the girls not to be aware of the guidelines. The main reason was the unavailability of the guidelines, and this made most respondents (80%) answer not applicable.

The participants gave several reasons for not washing hands, including a lack of water buckets at the toilets (28.1%), and about 23.1% said they did not wash hands because of a lack of hand washing soap. However, the majority of the participants explained that hand washing was not done because of both reasons: The lack of water buckets and soap for hand washing was 48.8%. At Chilukwa Primary School, school absenteeism of girls was 45%, and out of this, 20% was menstrual‐related.

### 3.3 Cultural Beliefs Surrounding Menstruation

The study found that some cultural beliefs surrounding menstruation did not allow girls to go to school due to fear of staining and shame to the family as well as the community. This was corroborated by what one of the respondents said:

During menarche, I was in house for 7 days without doing any household chores as our cultural settings did not allow me to attend classes in menses in fear to stain and is a taboo for boys to see menses that could only degenerate into l laughter (G4)

Another an adolescent girl on how poor sanitary facilities affected their performance, two of the respondents said these words;

we miss important lessons in class. When we come back to school, we fail to compete with our fellow pupils, especially when it is time for examinations. G 3

One respondent said:

Previously, when a girl attained the menarche was kept in the house for 7 days. The elder dressed the girl in a pad. The girl stayed with the soiled pad unchanged for 7days until no fresh blood was seen. On day 7, the soiled pad was laid under the mlama tree and buried it. MCG 2

In terms of whether there were problems associated with *this culture, one respondent said the following:*

there were problems such as odour because that was blood, and this made girls feel nauseated, and this brought loss of appetite. The other problem was skin laceration (chafing,) especially the thighs, due to the wet pad MCG4

## 4 Discussion

Menstruation is an important pubertal development. A lot of misconceptions about this process translate into poor knowledge and hygienic practices on menstruation. Adequate knowledge of menstruation and its hygienic practices would prevent reproductive ill‐health. This study aimed at exploring the menstrual hygiene practices among adolescent school girls in rural Mzimba, Malawi. Overall, results on demographic information showed that management of menstrual hygiene by respondents was in one way or the other affected by unavailability of sanitary facilities such as lack of hand washing places, lack of doors on gils toilet, improper disposal of menstrual waste and lack of MHM guidelines within the school authority. As such, the menarche was observed to cause fear in adolescent girls at primary schools, especially those with little or no knowledge about menstruation.

**Concerning availability of sanitary facilities at the school, the study** indicated the level of management of menstrual hygiene by schools was suboptimal. The school that had adequate sanitation facilities was expected to have fewer issues of MHM. Results indicated that Luhomero Primary School had 15% of the absenteeism of learners due to menstrual‐related issues, whereas at Chilukwa, 20% as indicated by the headmasters of both schools.

Both headmasters cited a lack of adequate sanitary facilities and the absence of essential facilities such as wash room for use by girls when soiled. This might directly affect the attendance of girls at school.

At both schools, the toilets were close to the boys’ toilets, and this compromised the privacy much needed for girls. In addition, the study found that all three toilets had no doors. This finding is higher than what was reported in Ethiopia, which found that only 25% of toilets had no doors [13]. Caruso et al. [14] found similar results that open toilets (no doors) do not provide privacy and dignity to the girls. Lack of private places to wash or change during menstruation at the school has strong implications to both health and education. Health‐wise it could lead to poor menstrual hygiene practices and subsequent UTIs. Furthermore, on the part of education, it could contribute towards absenteeism. The issue of privacy is very significant considering the cultural context, like Malawi, where menstruation is treated as a taboo and is not openly discussed.

In the study, it was that the sanitation facilities did not have water for hand washing after use. A similar finding was reported by Chidya et al. (2024) in rural Lilongwe, Malawi. Washing hands can keep pupils and the community healthy and prevent the spread of respiratory and diarrhoeal infections through contaminated hands with faecal matter. According to WHO [15], people are advised to wash hands to prevent germs that can spread from person to person or from surfaces to people when one has contaminated/dirty hands with faeces and if that one touches the eyes, nose and mouth with unwashed hands. Preparing or eating food and drinks with unwashed hands could cause diarrhoeal diseases because some parasitic eggs are invisible and spread fast via dirty hands.

The study also showed lack of policy guidelines regarding menstrual health hygiene at the schools, which could explain the unpleasant menstrual hygiene practices as demonstrated in lack of structures such as the aforementioned lack of hand washing equipment. Notably, a recent study conducted in Lilongwe suggested that Malawi is currently facing rapidly evolving menstrual practices, especially related to the use of materials and socioeconomic status, and how these interact with the notion of environmental sustainability (Alva‐Vidal 2022). The current shift to the use of disposable materials presents the court with a difficult puzzle to solve. The question arises: How do we ensure waste management for menstrual products when planning Wash and waste management strategies?

Previous studies found that about 44% of schools in Ethiopia had a changing room for pads [13]. In contrast, this study reported that although schools had a changing room for MHM, it was rarely used since using it meant someone was menstruating. This finding detects the effect of sociocultural values on MHM. In Malawi, menstruation is considered unclean and something that is not discussed freely; hence, girls would opt to stay home during menstruation. Nevertheless, for school girls, this could mean losing valuable school time and leading to absenteeism. Although this study did not assess the effect of menstruation on school achievement, previous scholars have alluded to the detrimental effect of menstruation on school education as it is directly linked to absenteeism and poor performance [16]. A study conducted in the neighbouring Tanzania found that found that lack of changing rooms in school was a significant predictor of school absenteeism among adolescent girls (AOR = 5.85 (95% CI: 4.82–7.93)) [17].

In this study a smaller proportion of participants used dust bins for disposing menstrual waste compared to 37% who used pad disposal dustbins in karonga district in Malawi [16]. The difference could be attributed to differences in availability of infrastructure at the different study sites. Nevertheless, menstrual hygiene practices in limited resource settings are very much influenced by the infrastructure [18].

In a study conducted by Nyasulu and colleagues in Karonga, about 60% of menstruators agreed that cultural practices regarding menstruation disturb their school activities [16]. Similarly, in these studies, it was indicated that girls were kept in the house for 7 days as part of culture, which affected school attendance. This finding is significantly pointing to the need to address cultural practices regarding menstruation to address education inequalities that may leave girls behind. Even though this paper did not assess the availability of menstrual education in schools, previous work indicates that more adolescents learn about menstruation from parents than teachers [16].

The results agreed with a study in North East Ethiopia in which 58% of girls reported that their school‐performance had declined after they had menarche. The study also indicated that school dropout was common among girls who experienced teasing and humiliation by classmates (Tegegne and Sisay 2014). Poor facilities for MHM may cause adolescent girls to remain at home until the menstruation is completed. Furthermore, as per culture indicated in this study, when girls are locked in the house for 7 days, they also lag behind as school hours are lost due to nursing menstruation.

### 4.1 Cultural Beliefs Surrounding Menstruation

In line with the study by Fehintola et al. (2017), it was also observed from the study that menstruation and menstrual practices were still clouded by taboos and sociocultural restrictions, resulting in adolescent girls remaining ignorant of the scientific facts and hygienic health practices, which sometimes resulted in adverse health outcomes. The fining of the study echoes the need to employ educational interventions to inform MHM practices in schools. Previous research demonstrated that after educational intervention, the hygiene behaviour and attitude among menstruating girls improve [19].

Using the bush was also at risk of polluting the environment, which could also facilitate the spread of gastrointestinal diseases such as cholera, shigellosis and dysentery. This might also expose the girl to snake bites and compromised privacy and dignity. The nongovernmental organisation (NGO)‐ Every Child in 2006 came and resolved some of the harmful taboos through community sensitisation on taboos which encouraged girls to be absent from school.

### 4.2 Strengths and Limitations

The main strength of the study is that it is the first of this kind in this district with previous studies focused on the urban area as such findings from the rural area remained unknown. The study is not without drawbacks. First, the study is limited in terms of advanced statistical analysis techniques and is limited to descriptive statistics without including inferential statistics, so it remains unknown if the study findings are statistically significant. Second, the study was limited to two schools in the rural area; hence, it cannot be generalised to other rural areas in the country. Third, the study sample size was relatively small to generalise to the entire population of Malawian adolescents in the rural area; hence, it is not representative of the country population. In addition, the study was conducted in one district; hence, it is not comparable to other districts in the country. Moreover, the data are prone to social desirability effect considering that it was subjective in nature.

## 5 Conclusion

The study has revealed unfavourable conditions related to unavailability of sanitary facilities such as hand washing facilities, changing room for girls and lack of MHM guidelines. The poor state of available sanitary facilities such as lack of bathrooms to wash when soiled, lack of lockable toilets compromise the privacy of young adolescents who are not allowed dignity in social cultural setting where menstruation is treated as a taboo. Practical implications for policy and practice are required.

The study results imply that there is need for ministry of education and local government to formulate policy to ensure guidelines for MHM in schools and the required infrastructure such as water and soap available for hand washing. Furthermore, schools need to focus on privacy and safety of girls by ensuring availability of lockable changing rooms at primary school in the area for adolescents to have dignity and promote empowerment among these young girls. The study also ensures MHM education is included in the curriculums to ensure safe and hygienic practices are adopted and practiced by young adolescents in the area.

## Author Contributions

**Benard C. G. Kamanga**: conceptualisation, investigation, methodology, formal analysis, supervision, writing – review and editing, writing – original draft. **Zondiwe Chingati**: investigation, writing – original draft, writing – review and editing, methodology, formal analysis. **Thokozani Mzumara**: writing – original draft, writing – review and editing, methodology, formal analysis, investigation. **Sellah Vitumbiko Nkowani**: conceptualisation, methodology, formal analysis, visualisation, investigation, writing – original draft, writing – review and editing .

## Funding

The authors have nothing to report.

## Ethics Statement

The study was ethically approved by the University of Livingstonia Research Committee (UNILIA‐REC/PGS/01/23) on fourth September 2023 and adhered to the declarations of Helsinki.

## Consent

The study obtained informed consent.

## Conflicts of Interest

The authors declare no conflict of interest.

## Data Availability

The data are available upon reasonable request.
